# Supplementary material for: Development and evaluation of an ARTIC-based amplicon sequencing assay for whole-genome characterization of respiratory syncytial virus
Source: J Clin Microbiol. 2026 Sep 3;64(10):e00468-26. doi: 10.1128/jcm.00468-26 (PMC13712889; doi:10.1128/jcm.00468-26)
Supplement: Supplementary file 1 [file jcm.00468-26-s0001.docx]

**SUPPLEMENTAL FIGURES AND TABLES LEGEND**

**Table S1**. A total of 102 RSV-A and 112 RSV-B clinical samples were sequenced. 75 out of 102 RSV-A samples passed while 75 out of 112 RSV-B samples passed the validation acceptance criteria. Whole-genome sequencing was compared to the reference method, Thermo Fisher Respiratory Viral Panel PCR assay. Mean depth and percent genomic coverage were evaluated. *Please refer to the Table_S1 sheet in the Excel file.*

**Table S2**. The lower limit of detection (LOD) for RSV-A and RSV-B was determined by an initial test of finding the estimated LOD with serial dilutions (A). Replicates LOD confirmation for RSV-A (B) and RSV-B (C). *Please refer to the Table_S2 sheet in the Excel file.*

**Table S3**. Mixtures of RSV-A and RSV-B reference material were used to evaluate multiplexing and ability to detect multiple targets with the WGS assay. All mixes passed validation acceptance criteria. 14/15 (93%) of replicates had majority of the mapped reads aligned to the correct target reference genome. *Please refer to the Table_S3 sheet in the Excel file.*

**Table S4**. A variety of 31 non-RSV samples was sequenced to evaluate specificity and cross-reactivity of the WGS assay as part of the validation. All samples did not have any positive kmer detection hits for both RSV-A and RSV-B. *Please refer to the Table_S4 sheet in the Excel file.*

**Table S5**. Pairwise nucleotide differences between duplicates of the same sample within the same sequencing run by one operator for intra-run precision (repeatability) among 16 high-quality RSV-A and RSV-B samples that passed validation acceptance criteria. Each pair of consensus sequences were nearly 100% identical with 14 out of 16 samples had no more than 3 nucleotide differences. *Please refer to the Table_S5 sheet in the Excel file.*

**Table S6**. Pairwise nucleotide differences between 3 operators for inter-run precision (reproducibility) among 53 high-quality RSV-A and RSV-B samples that passed validation acceptance criteria. Each pair of consensus sequences were nearly 100% identical with 0 to 5 nucleotide differences. *Please refer to the Table_S6 sheet in the Excel file.*

**Table S7**. Pairwise nucleotide differences between 3 separate sequencers (2 NextSeq 1000s, 1 NextSeq 2000) for inter-run precision (reproducibility) among 57 high-quality RSV-A and RSV-B samples that passed validation acceptance criteria. Each pair of consensus sequences were nearly 100% identical with 0 to 4 nucleotide differences. *Please refer to the Table_S7 sheet in the Excel file.*

**Table S8.** GISAID Accession IDs for 151 submitted complete RSV genomes. *Please refer to the Table_S8 sheet in the Excel file.*

**SUPPLEMENTAL FIGURES**

**Figure S1.** Heatmap of (**A**) RSV-A (**B**) RSV-B showing the median depth of coverage per amplicon (x-axis) for samples meeting validation acceptance criteria (y-axis) using the PrimalScheme designed custom primers.

A

B

**Figure S2.** Heatmaps of RSV-A (**A**) and RSV-B (**B**) showing the median depth of coverage per amplicon (x-axis) across samples that met validation acceptance criteria (y-axis) using ARTIC primers.

A

**B**
